# Snake Venom Metalloproteinases and Their Peptide Inhibitors from Myanmar Russell’s Viper Venom

**DOI:** 10.3390/toxins9010015

**Published:** 2016-12-30

**Authors:** Khin Than Yee, Morgan Pitts, Pumipat Tongyoo, Ponlapat Rojnuckarin, Mark C. Wilkinson

**Affiliations:** 1 Faculty of Medicine, Chulalongkorn University, Bangkok 10330, Thailand; khinthanyee@gmail.com (K.T.Y.); kpsppto@ku.ac.th (P.T.); 2 Institute of Integrative Biology, University of Liverpool, Liverpool L69 7ZB, UK; morganpitts@hotmail.co.uk

**Keywords:** snake venom metalloproteinases, snake venom metalloproteinase inhibitors, Russell’s viper, viper venom

## Abstract

Russell’s viper bites are potentially fatal from severe bleeding, renal failure and capillary leakage. Snake venom metalloproteinases (SVMPs) are attributed to these effects. In addition to specific antivenom therapy, endogenous inhibitors from snakes are of interest in studies of new treatment modalities for neutralization of the effect of toxins. Two major snake venom metalloproteinases (SVMPs): RVV-X and Daborhagin were purified from Myanmar Russell’s viper venom using a new purification strategy. Using the Next Generation Sequencing (NGS) approach to explore the Myanmar RV venom gland transcriptome, mRNAs of novel tripeptide SVMP inhibitors (SVMPIs) were discovered. Two novel endogenous tripeptides, pERW and pEKW were identified and isolated from the crude venom. Both purified SVMPs showed caseinolytic activity. Additionally, RVV-X displayed specific proteolytic activity towards gelatin and Daborhagin showed potent fibrinogenolytic activity. These activities were inhibited by metal chelators. Notably, the synthetic peptide inhibitors, pERW and pEKW, completely inhibit the gelatinolytic and fibrinogenolytic activities of respective SVMPs at 5 mM concentration. These complete inhibitory effects suggest that these tripeptides deserve further study for development of a therapeutic candidate for Russell’s viper envenomation.

## 1. Introduction

Russell’s viper (*Daboia russelii*) is a medically important snake, variants of which are distributed throughout East and Southeast Asia. A Russell’s viper bite has a 60% morbidity rate and the fatality rate is 8.2% in Myanmar [1]. The cause of death includes shock, massive bleeding and renal failure. Snake venom metalloproteinases (SVMPs) play a major role in the local and systemic clinical manifestations: blistering, necrosis and bleeding from the fang marks and incoagulable blood, thrombocytopenia, spontaneous systemic bleeding, hypotension, increased permeability and reduced urine output [2]. Although Russell’s viper antivenoms are available, their efficacy in reversal tissue damage, such as acute renal failure, is limited [3]. Novel treatment modalities are required.

Snake venom metalloproteinases (SVMPs) play major roles in pathogenesis of Russell’s viper bites [4,5]. SVMPs are categorised into P-I to P-III classes according to their domain organization with different molecular weights [6]: Class I (P-I) contains only a prodomain and a metalloproteinase domain (20–30 kDa); Class II (P-II) contains a prodomain, metalloproteinase domain followed by disintegrin domain (30–60 kDa); Class III (P-III) contains a pro, metalloproteinase, disintegrin-like and cysteine-rich domain (60–100 kDa). There are subclasses in P-II and P-III depending on post-translational modifications. The variation in domain composition between SVMP classes contributes to a wide spectrum of substrate specific proteolytic activity. The active site of the metalloproteinase domain has a consensus H^142^EXXHXXGXXH^152^ sequence. The catalytic zinc-ion is located at the bottom of the active-site cleft, and tetrahedrally coordinated by His^142^, His^146^, His^152^, and a water molecule anchored to Glu^143^ [7]. The degradation of endothelial cell membrane proteins (integrin and cadherin), basement membrane components (fibronectin, laminin and collagen) and blood coagulation proteins (fibrinogen, factor X and prothrombin) leads to haemorrhage. Generally, P-III SVMPs have more potent haemorrhagic activity than P-I and P-II SVMPs [8]. 

In order to protect against auto-digestion by SVMPs, snake venom of several species are found to contain natural protease inhibitors: citrate and small peptides. The latter bind selectively to SVMPs in the venom glands to protect glandular tissues and venom factors from self-digestion by SVMPs [9]. Three endogenous peptides: pyroGlu-Lys-Trp (pEKW), pyroGlu-Asn-Trp (pENW) and pyroGlu-Gln-Trp (pEQW) isolated from venom of Taiwan habu (*Trimeresurus mucrosquamatus*) showed an inhibitory action on proteolytic activity of metalloproteinases present in the crude venom [10]. It is reported that these peptide inhibitors regulate the proteolytic activities of their SVMPs in a reversible manner under physiological conditions [11]. Other pit vipers, such as *Bothrops asper* [12] and some rattlesnakes [13], also have venoms containing endogenous tripeptides: pEQW and pENW. African vipers, *Echis ocellatus* and *Cerastes cerastes cerastes*, have pEKW tripeptides. These tripeptides are encoded by tandemly repeating elements from the transcripts which also contain a CNP (C-type natriuretic peptide) homologous sequence at the C-terminus [14]. Two peptides: PtA (pENW) and PtB (pEQW) isolated from venom liquor of *Deinagkistrodon acutus* (Hundred-pacer viper) showed anti-human platelet aggregation activity in vitro and protection effects on ADP-induced paralysis and formation of pulmonary thrombosis in mice [15].

We hypothesized that Myanmar Russell’s viper venom might contain endogenous peptides to neutralise its own potent SVMPs. The goal of this research was to purify and identify specific SVMP inhibitors (SVMPIs) from the venom as well as from venom glands and to determine their inhibitory action on purified SVMPs from same source of venom. From the transcriptome of the snake, novel SVMPI transcripts containing tripeptide motifs and ANP (atrial natriuretic peptide) sequences were found. Two tripeptides were purified from the venom and identified as pERW and pEKW. Their effect on biological activities of two SVMPs: RVV-X and Daborhagin from the same venom, purified through newly developed strategy, were examined. Both synthetic peptides showed complete inhibitory action on the gelatinolytic activity of RVV-X and fibrinogenolytic activity of Daborhagin at 5 mM concentration (approximate protease to inhibitor molar ratio of 1:500). The results might contribute to the development of complementary candidates for current antivenom therapy of Russell’s viper bites, as well as for novel therapeutic agents for cardiovascular diseases.

## 2. Results

### 2.1. Purification and Identification of SVMPs from Myanmar Russell’s Viper Venom

#### 2.1.1. Purification of SVMPs

**Figure 1:**
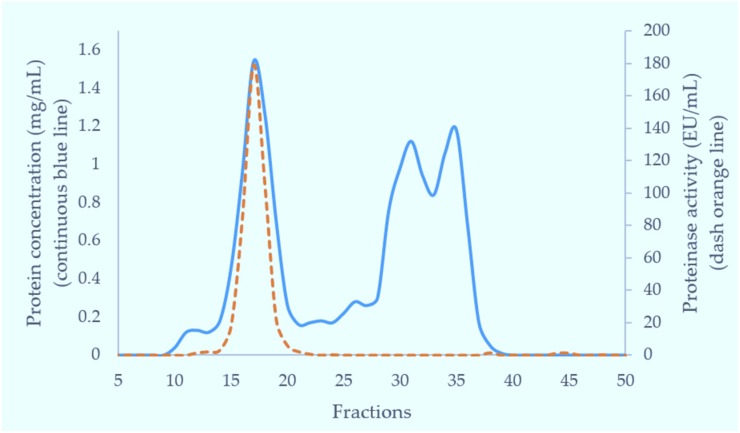
Fractionation of Myanmar Russell’s viper crude venom through Superdex 200 gel filtration column (5 × 160 cm). Crude venom was separated in 0.01 M phosphate buffered saline (pH 7.4) at 2 mL/min. Each fraction was 6 mL in volume. The blue continuous line shows the protein concentration (mg/mL) and the orange dashed line shows protease activity (EU/mL) in collection fractions.

**Figure 2:**
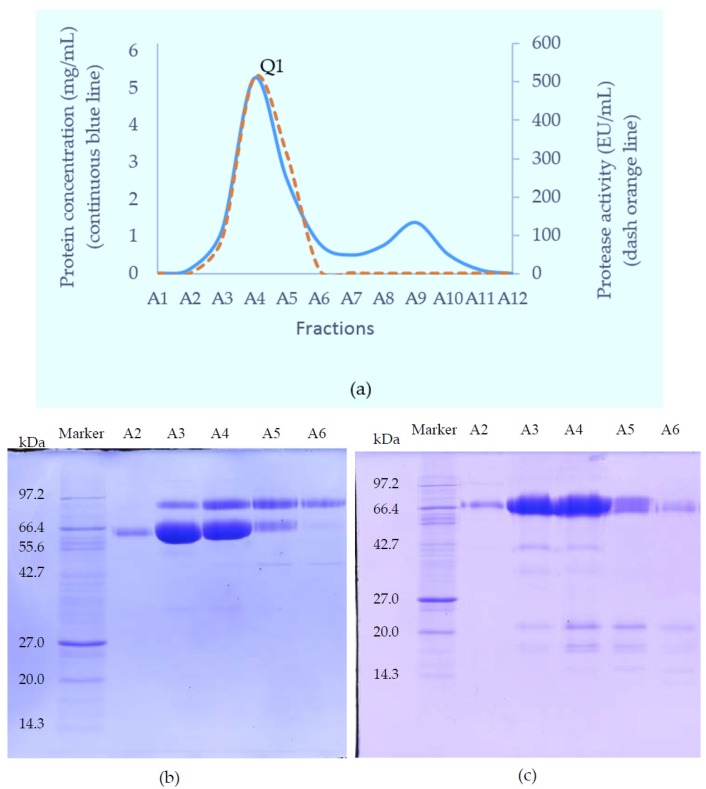
Separation of fractions 15–18 from GFC on a Resource Q anion-exchange column (**a**) Chromatography trace showing protein concentration and caseinolytic activity. Peak one (Q1) contained fractions with protease activity; SDS-PAGE of the purified proteins under (**b**) non-reducing; and (**c**) reducing conditions.

The crude venom of Myanmar Russell’s viper (MRV) was initially separated on a Superdex 200 column. Of the three major protein-containing peaks, only the first possessed caseinolytic activity (Figure 1). These fractions were pooled and further purified on a Resource Q anion-exchange column. The proteins resolved into two peaks and the first peak (Q1) exhibited caseinolytic activity (Figure 2a). The purity of proteins in Q1 was determined on both reducing and non-reducing SDS-PAGE. Non-reducing SDS-PAGE of this fraction showed it to contain two bands at 85 kDa and 67 kDa. Under reducing conditions, the main protein bands ran at approximately 67 kDa band and low molecular weight (15–20 kDa) bands were evident.

**Figure 3:**
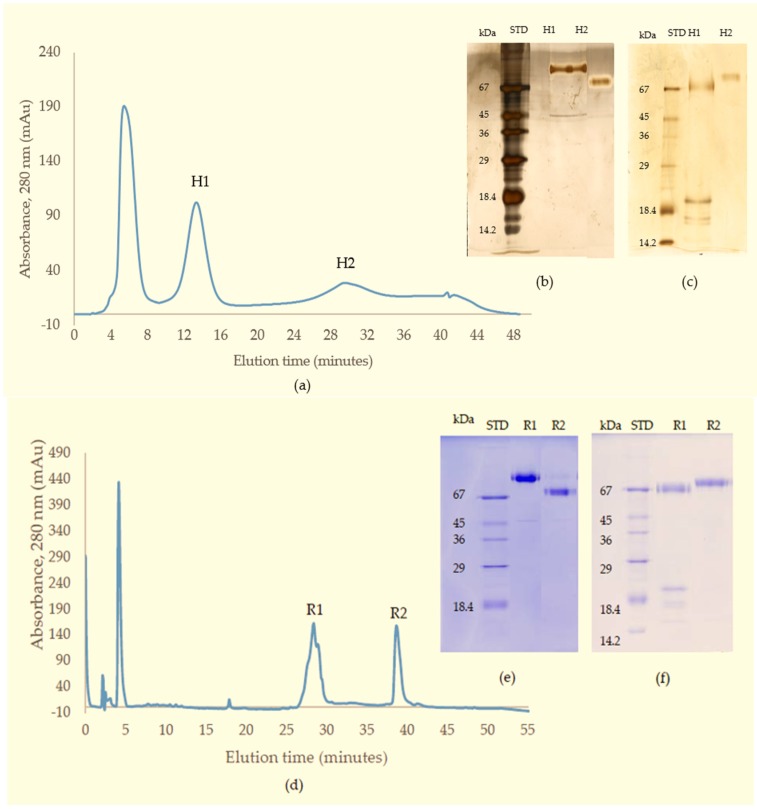
(**a**) Chromatography of fraction Q1 from the Resource Q column on Phenyl Superose column (HIC) showing protein-containing peaks (H1 and H2); (**b**) non-reducing; and (**c**) reducing SDS-PAGE of purified proteins (silver-stained); (**d**) Chromatography of fraction Q1 from Resource Q column on a C4 RP-HPLC column; Two protein peaks were observed: R1 and R2; SDS-PAGE of purified proteins R1 and R2 (**e**) under non-reducing conditions; and (**f**) reducing conditions.

This material (Q1) was then subjected to further separation on either HIC for activity studies, or RP-HPLC when proteins were prepared for mass spectrometry. A Phenyl Superose column was used for HIC during which the protein fraction resolved into 2 peaks: H1 (eluted at 13 min), and H2 (eluted at 29 min), respectively (Figure 3a–c). For RP-HPLC, a Phenomenex Luna C4 column was used and again the proteins were separated into 2 peaks (R1 and R2) (Figure 3d–f). SDS-PAGE analysis and activity studies showed H1 to be the same protein as R1 running at 85 kDa under non-reducing conditions, but at 67 kDa with several subunits at 15–20 kDa when reduced. H2 is the same as R2, with a single band at 68 kDa under both reducing and non-reducing conditions.

#### 2.1.2. Identification of SVMPs

**Figure 4:**
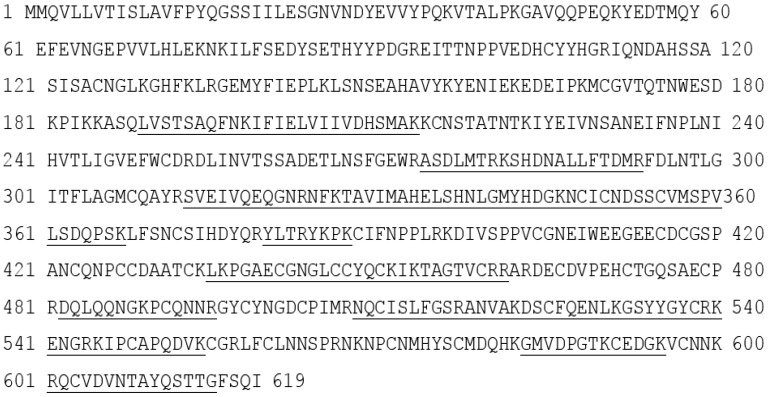
Data from LC-ESI-MS/MS analysis of the tryptic peptides from purified RVV-X. The data was obtained by digesting the R1 fraction from RP-HPLC with trypsin. The prepro-sequence of RVV-X H chain (VM3CX_DABSI; Q7LZ61) annotated to show the peptides (underlined) identified in this analysis. All matched peptides were found within the sequence of the processed protein (residues 189–615).

Both proteins with protease activity were purified by C4 RP-HPLC in preparation for mass spectrometric analysis (see R1 and R2 in Figure 3d). For R1, the protein was reduced and treated with iodoacetamide and digested with trypsin in the presence of 2 M urea and the digest was analysed using LC-ESI-MS/MS (Figure 4). LC-ESI-MS/MS analysis of the tryptic peptides provided sufficient sequence coverage to match to the mature sequence (residues 189–615) of the Eastern Russell’s viper (*Daboia russelii siamensis*) RVV-X H chain VM3CX_DABSI (Q7LZ61) (Figure 4). In the same digest mixture we also found matches to RVV-X light chain proteins LC1 SLLC1_DABSI (Q4PRD1) and LC2 SLLC2_DABSI (Q4PRD2) from the same species (data not shown).

**Figure 5:**
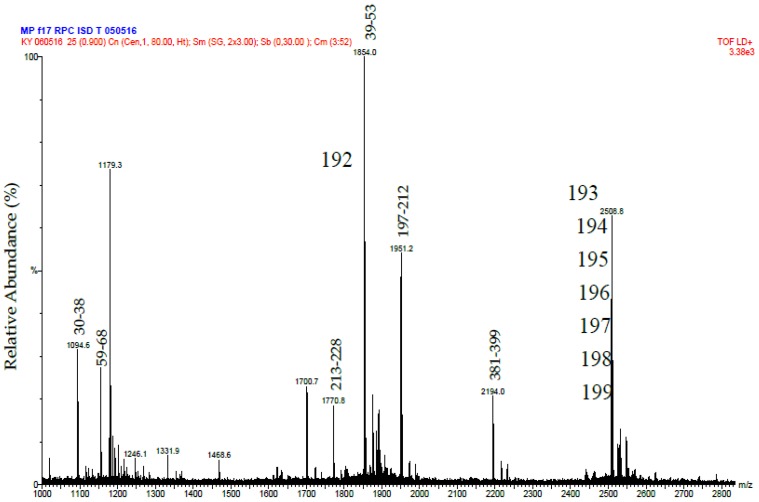
MALDI-MS spectrum of tryptic peptides from purified Daborhagin. The data was obtained by digesting the R2 fraction from RP-HPLC with trypsin. The numbers above the peptides masses indicates the residue numbers for the peptides matched to the sequence of Daborhagin K (VM3DK_DABRR) (B8K1W0). All *m*/*z* values are for the M + H^+^ ions. The ions at 1854 and greater have been labeled with the *m*/*z* value for the ion containing one carbon as the C^13^ isotope.

The protein R2 from RP-HPLC was digested in the same way as R1. In this case MALDI-MS analysis was used to identify tryptic peptides that matched the mass [M + H^+^] of those predicted from the sequence of Daborhagin-K (Indian Russell’s viper) (VM3DK_DABRR) (B8K1W0) (Figure 5). The majority of the most abundant peptides matched the mass of expected tryptic peptides and notably we found many of the same tryptic peptides as did Chen et al. (Table 2 in ref. [16]) for Daborhagin-M.

As a result of this work we can identify R1, H1 as the Myanmar Russell’s viper RVV-X and R2, H2 as Myanmar Russell’s viper Daborhagin and will to refer them as such from hereon.

### 2.2. Analysis of SVMPI Transcripts from Myanmar Russell’s Viper Transcriptome

**Figure 6:**
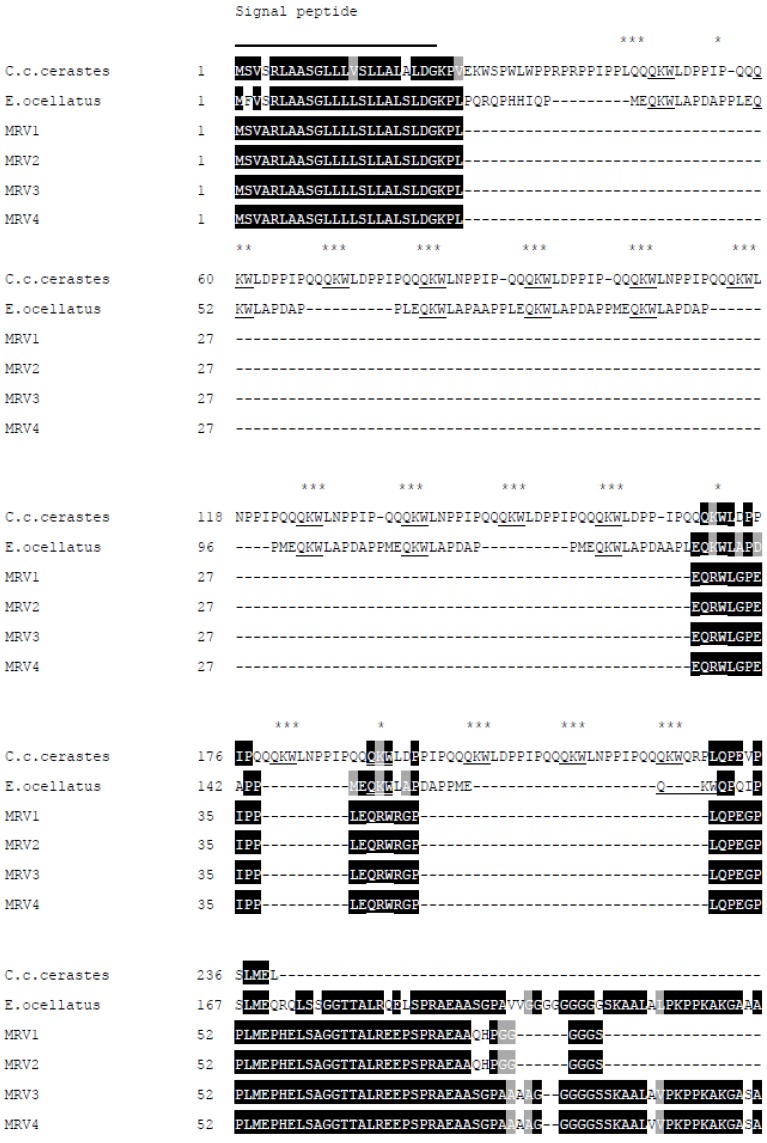

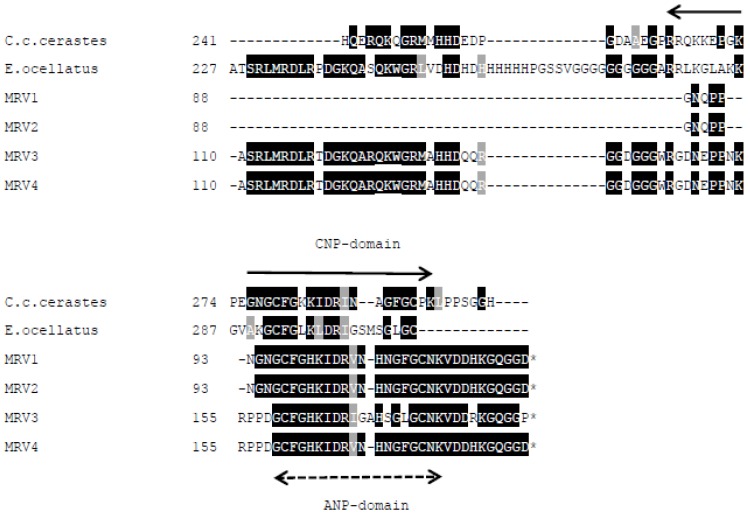
Multiple sequence alignment of the polypeptide encoded by Myanmar Russell’s viper SVMPI transcripts (MRV1-4) with those of two African vipers [*C. c. cerastes* (A8YPR9) and *E. ocellatus* (A8YPR6)]. The signal peptides are denoted by a solid line. The active tripeptides are underlined and identified with three asterisks. The varied residue is identified by a single asterisk. The CNP domains are indicated with a solid arrowed line and ANP domains with a dashed arrowed line.

From the transcriptome of Myanmar Russell’s viper venom glands, a total of 4 contigs were annotated as the Snake Venom Metalloproteinase Inhibitors (SVMPI). The conceptually translated proteins were aligned with those transcripts of African vipers, *Echis ocellatus* (A8YPR6) and *Cerastes cerastes cerastes* (A8YPR9). The signal peptides are highly similar and a new tripeptide QRW motif in addition to a QKW motif was found in the MRV transcripts. The tripeptides were flanked by the conserved PXXQ(K/R)WXXP motifs. The SVMPI transcripts of MRV also contained a conserved poly-Gly (pG) motif instead of the poly-His poly-Gly (pHpG) seen in *E. ocellatus* SVMPI transcripts. Moreover, the C-terminal portion of the SVMPI transcripts of MRV have an atrial natriuretic peptide (ANP) domain in place of the C-type natriuretic peptide (CNP) domain in the two African viper SVMPI transcripts (Figure 6).

### 2.3. Purification and Identification of Tripeptides

**Figure 7:**
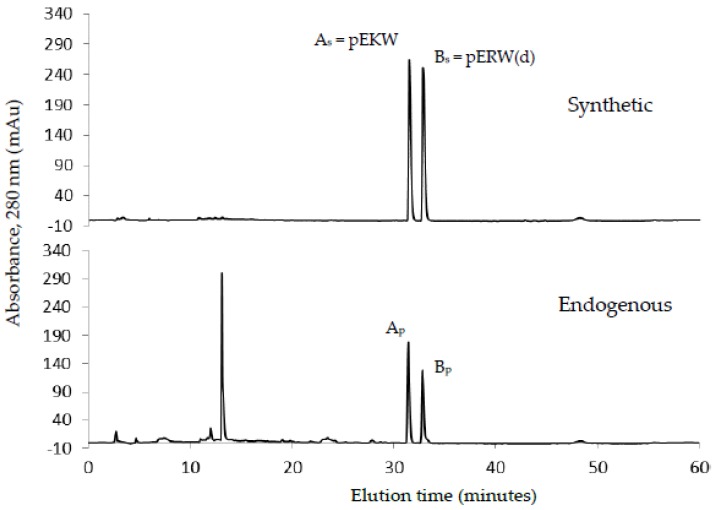
C18 RP-HPLC analysis of synthetic tripeptides, pEKW and pERW (1 μg of each) (**upper panel**); and fraction 48 (200 μL) (**lower panel**) obtained from gel filtration chromatography of crude venom. Peak A_s_ and peak B_s_ represent the two synthetic tripeptides. Peak A_p_ and peak B_p_ represent the two tripeptides from Fraction 48.

**Figure 8:**
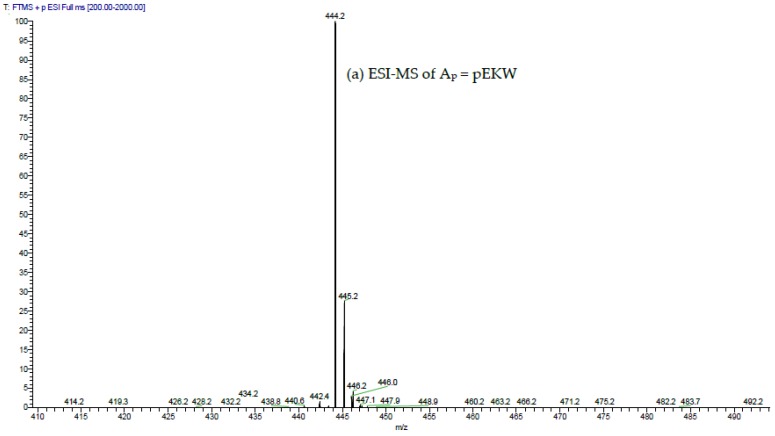

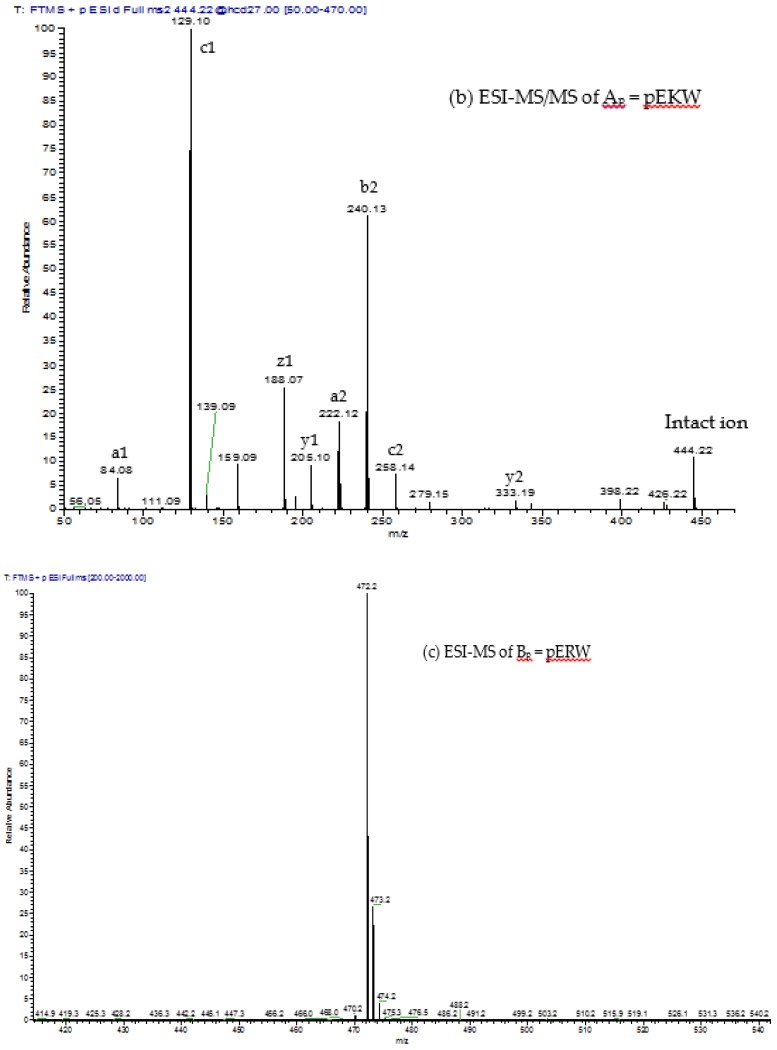

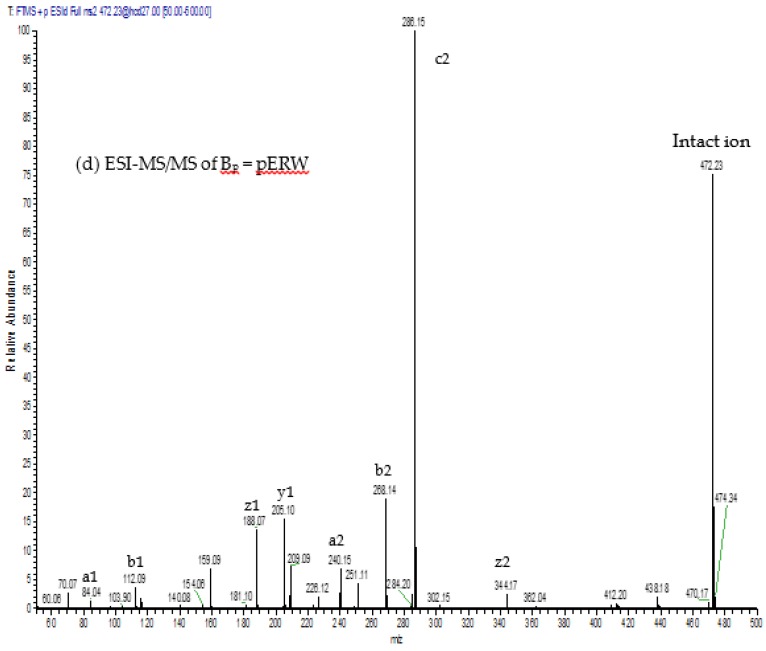
ESI-MS and ESI-MS/MS spectra of (**a**,**b**) peak A_p_; and (**c**,**d**) peak B_p_ isolated via RP-HPLC of low molecular material obtained from GFC of crude MRV venom; (**a**,**c**) ESI-MS spectra. The values indicated are for the M + H^+^ ions. These are within 0.05 Da of the predicted values for pEKW and pERW (monoisotopic masses are 443.2 and 471.2 respectively); (**b**,**d**) ESI-MS/MS spectra. The predicted a, b, c, y and *z* ions are indicated above the mass values.

The low molecular fractions from Superdex 200 chromatography were analysed using C18 RP-HPLC. Fraction 48 was found to contain the highest concentration of the tripeptides. Upon RP-HPLC analysis of this fraction, two peaks (A_p_ and B_p_) eluted close together at 31–33 min (Figure 7). These peaks possessed the same elution time as that of two synthetic peptides pEKW (peak A_s_) and pERW (peak B_s_), respectively. RP-HPLC analysis of mixtures of natural and synthetic tripeptides showed perfect co-chromatography. The purified endogenous tripeptides were then analysed using ESI-MS. The resultant spectra of peak A_p_ showed a strong M + H^+^ ion at *m*/*z* 444.2, (the predicted monoisotopic mass of pEKW is 443.2). Analysis of peak B_p_, also showed a strong M + H^+^ ion at *m*/*z* 472.2(the predicted monoisotopic mass of pEKW is 471.2) (Figure 8). MS/MS analysis of these tripeptides produced a set of fragment ions consistent with their expected amino acid sequence (data not shown).

### 2.4. Characterization of RVV-X and Daborhagin

The purified proteins RVV-X and Daborhagin from HIC were used for characterization of their gelatinolytic and fibrinogenolytic activities. Using a caseinolytic assay, both proteins were shown to be completely inhibited with metal chelators such as EDTA, 1,10-phenanthroline and citrate. 

**Figure 9:**
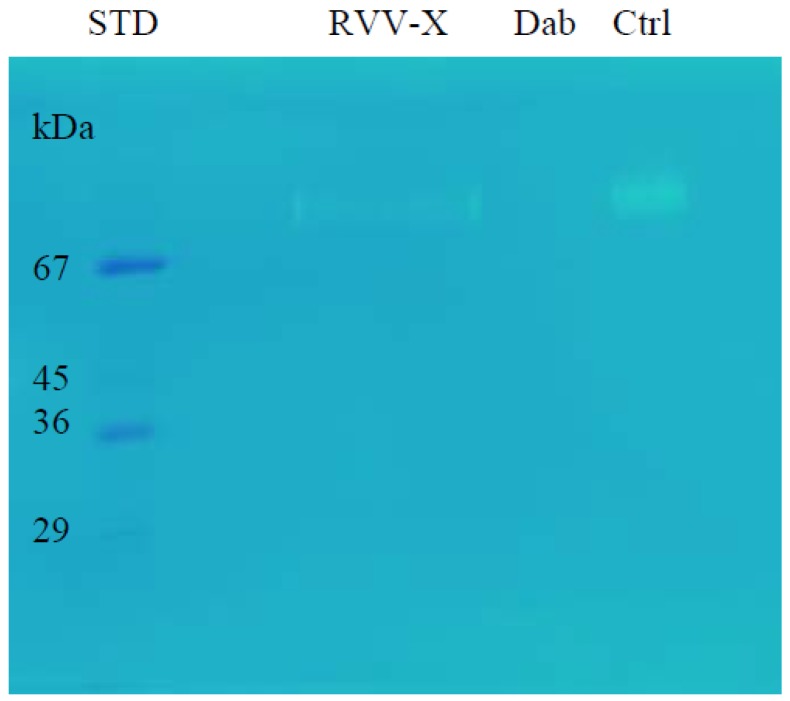
Gelatinolytic activity of RVV-X and Daborhagin on 0.25% gelatin zymogram after 48 h-incubation at 37 °C. Dab: Daborhagin; Ctrl: combined sample of two purified proteins. RVV-X, but not Daborhagin, showed gelatinolytic activity.

The gelatinolytic activity was analysed by zymography. On the gelatin zymogram (0.25% gelatin), RVV-X showed a clear band but Daborhagin did not show any gelatin degradation (Figure 9). 

**Figure 10:**
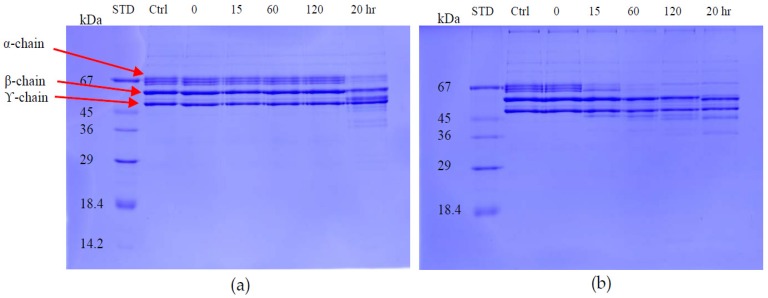
Fibrinogenolytic activity of RVV-X and Daborhagin. 10 μg/mL purified enzyme was incubated with 1 mg/mL fibrinogen solution at 0, 15, 60, 120 min and 20 h-incubation. Sample: (**a**) RVV-X; (**b**) Daborhagin. Ctrl: fibrinogen control; STD: molecular weight markers.

The fibrinogenolytic activity of the two proteins was determined using 12% SDS-PAGE after incubation with fibrinogen solution for different times at 37 °C. Daborhagin digested the α-chain of human fibrinogen within 1 h of incubation. RVV-X only revealed fibrinogenolytic activity after an overnight incubation (Figure 10).

### 2.5. Inhibitory Assay with Synthetic Tripeptides

#### 2.5.1. Effect of Synthetic Tripeptides on the Gelatinolytic Activity of RVV-X

**Figure 11:**
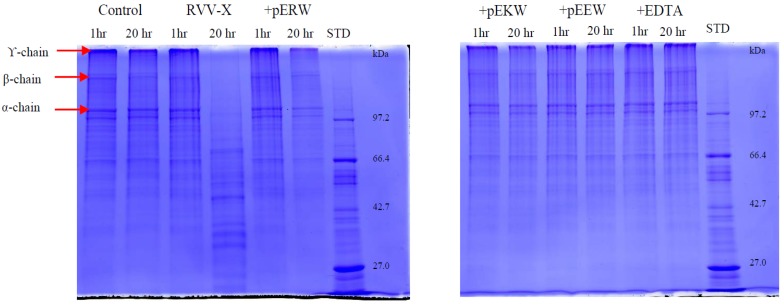
Gelatinolytic activity of RVV-X. Gelatin (1 mg/mL) was incubated with 10 μg/mL RVV-X for 1 h and 20 h at 37 °C, either with or without EDTA or synthetic tripeptides: pERW, pEKW, pEEW. Control = reduced gelatin, STD = molecular weight markers.

The gelatinolytic activity of RVV-X was completely inhibited by both synthetic tripeptides pEKW and pERW at 5 mM concentration when incubated with 1 mg/mL gelatin solution at 37 °C (Figure 11). The α-chains (100 kDa & 130 kDa), β-chain (200 kDa) and γ-chain (300 kDa) of gelatin were totally degraded by RVV-X in a 20 h-incubation, whereas these gelatin subunits were still intact in samples containing tripeptides or EDTA after 20 h of incubation. The tripeptide pEEW was included in the assay to test the specificity of amino acid residue in the second position of the tripeptides.

#### 2.5.2. Effect of Synthetic Tripeptides on the Fibrinogenolytic Activity of Daborhagin

**Figure 12:**
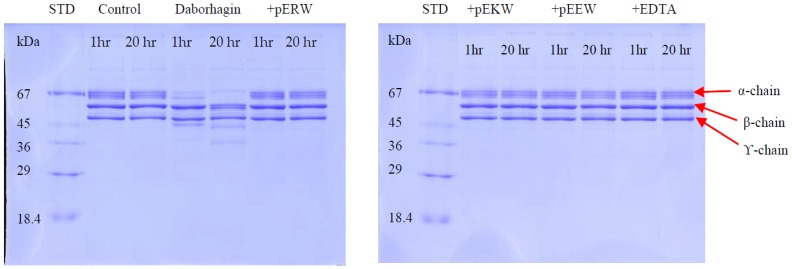
Fibrinogenolytic activity of Daborhagin. Fibrinogen (1 mg/mL) was incubated with 10 μg/mL Daborhagin for 1 h and 20 h at 37 °C with or without EDTA or synthetic tripeptides: pERW, pEKW, pEEW. Control = reduced fibrinogen, STD = molecular weight markers.

The fibrinogenolytic activity of Daborhagin was completely inhibited by both synthetic tripeptides pEKW and pERW at 5 mM concentration when incubated with 1 mg/mL fibrinogen at 37 °C (Figure 12).

## 3. Discussion

In the current study, we have developed a new method which can be used to simultaneously isolate the two SVMPs, RVV-X and Daborhagin, from Myanmar Russell’s viper venom. The relative amounts of these enzymes in the venom were determined. In addition, four novel RNA sequences of SVMP inhibitor (MRV1-4) were derived from the venom gland transcriptome. These sequences are different from those of previously reports in other snakes. For the first time in studies of Russell’s viper venom, two tripeptide SVMP inhibitors, pERW and pEKW have been isolated. Evidence for the complete inhibition of RVV-X and Daborhagin activities by these tripeptides is presented to support our hypothesis.

Russell’s viper is a venomous species of the South-East Asian region. The clinical manifestations of its bites reflect the high content of proteases such as snake venom serine proteases and snake venom metalloproteinases (SVMPs). It has been shown that the SVMPs comprise approximately 11% to 65% of the total protein in the Viperidae venoms [17]. In Myanmar Russell’s viper, SVMPs contribute to 20% of the crude venom (data not shown) and Class III SVMPs are found to be the major component. In comparison with other species, the Myanmar species have 6–7 times more Daborhagin than Indian species [16] and SVMPs, mainly RVV-X, in Sri Lankan species comprise just 6.9% of the crude venom [18]. Thus, it can be noted that the Myanmar venom contains greater amounts of SVMPs than that of the Indian and Sri Lankan species. The variations in types and amounts of SVMPs in venom among different subspecies of Russell’s viper might be due to diversity in their prey at different locations and this could lead to the dissimilar severity or clinical presentations of snakebite patients.

Russell’s viper venom factor X activator (RVV-X) is a well-characterised Class III metalloproteinase (formally known as Class IV) which specifically activates coagulation factor X by hydrolysis of an Arg-Ile bond in factor X. It is a glycoprotein consisting of a heavy chain (α-chain, 57.6 kDa) and two light chains (β- and γ-chains, 19.4 kDa and 16.4 kDa) linked by disulfide bonds [19]. In addition to proteolytic activity on factor X and IX, RVV-X also inhibits collagen- and ADP-stimulated platelet aggregation [20] and has a strong affinity for protein S [21]. Factor X activators are also found in *Vipera lebetina* (blunt-nosed viper) in which it exhibits specific proteolytic activity towards human factor X and also factor IX, but it is not active against prothrombin nor fibrinogen [22].

In the present study, the purified RVV-X was shown to be composed of a heavy chain (67 kDa) and two light chains (20 kDa and 15 kDa). The two thin bands on SDS-PAGE at around 15 kDa level suggested that the γ-light chain in Myanmar species might exist as 2 forms, likely due to either amino acid variation or differences in *N*-glycosylation. Our experiments showed that MRV RVV-X possesses hydrolytic activity to gelatin (Type I collagen, bovine), which had not been characterised before for RVV-X.

Another potent Class III SVMP, Daborhagin, composed of metalloproteinase, disintegrin and cysteine-rich domains, was also purified from MRV venom. The Daborhagin-M from Myanmar Russell’s viper venom specifically digested the α-chain of fibrinogen, fibronectin and type IV collagen in vitro and exhibited haemorrhagic [16], edema inducing and myonecrotic activity in mice [23]. In our studies, a 67 kDa metalloproteinase was isolated and matched to Daborhagin-K from Indian species using mass spectrometric analysis of tryptic peptides. This MRV Daborhagin exhibited potent α-fibrinogenolytic activity, but did not digest gelatin.

In the current purification strategy, the two SVMPs were co-purified initially, but then could be separated from each other using either hydrophobic interaction chromatography or RP-HPLC. Better resolution was evident on RP-HPLC, and the presence of multiple forms of RVV-X was indicated by the irregularity of the RVV-X RP-HPLC peak, suggesting heterogeneity of the protein (R1, Figure 3d). Two isoforms of the heavy chain and 6 isoforms of the light chain from RVV-X have been revealed on 2-D electrophoresis in the proteomic study of Risch, M et al. in the same species [24]. 

New SVMPI transcripts from Myanmar Russell’s viper were discovered containing novel two inhibitory tripeptides, QKW and QRW. The tripeptide sequences are found in the same transcript as natriuretic peptide sequences, as is the case in African vipers. This assortment of different peptide sequences in the same transcript could be related to independent evolution of toxin genes in snakes. The conserved proline residues in the consensus sequence PXXQ(K/R)WXXP might be a signal point for cleavage of tripeptides from transcripts. The mechanism for release of tripeptides from their transcripts is still unknown. These tripeptides and natriuretic peptides are observed separately in venom, although they are encoded from the same transcript. Since ANP is homologous to hormone, it might be processed near the effective cells. The release and modification of tripeptides [25] might probably occur during the exocytosis process at an earlier stage than the natriuretic peptides [26].

The aforementioned inhibitory tripeptides were purified from the MRV venom as their pyroglutamate forms, pEKW and pERW. These were identified using RP-HPLC (co-chromatography with synthetic peptides), LC-ESI-MS analysis of intact mass and LC-ESI-MS/MS sequencing. Although the tripeptide pEKW purified from MRV venom has been found in other snake species, such as *Trimeresurus mucrosquamatus* [10], *Echis ocellatus*, and *Cerastes cerastes cerastes* [14], the tripeptide pERW purified here has not been found in the venom of any other snake species.

The synthetic tripeptides pERW, pEKW and pEEW showed complete inhibition of the gelatinolytic activity of RVV-X and of the fibrinogenolytic activity of Daborhagin at 5 mM concentration of each inhibitor. Non-selective inhibition of all three synthetic peptides on biological activities of SVMPs reflects the importance of the first pyroGlu and the final tryptophan residue in the blocking mechanism at the active site of SVMP. The crystal structure of TM-3 (a SVMP from *Trimeresurus mucrosquamatus*) bound to tripeptide inhibitors (a proteinase and inhibitors model from Taiwan habu) revealed that the inhibitor Trp residue deeply inserts into the S-1 pocket of the protease and provides a greater inhibition than other smaller amino acids. Similarly, the pyro-ring of the inhibitor is required for fitting into the S-3 position of the protease and the activity of inhibitor becomes weaker in the absence of pyro-ring. The native middle residue is also position-specific to the S-2 site [11]. Tripeptides from different species share same first (pyroGlu-) and third (tryptophan) residues. The variability of the middle residue may be dependent on species variation of the SVMPs.

## 4. Conclusions

In summary, we have isolated and identified two major SVMPs and two endogenous tripeptides from Myanmar Russell’s viper venom. The two synthetic tripeptides showed specific inhibition against the fibrinogenolytic and gelatinolytic activities of the SVMPs. These findings may provide a means to explore potential drug design in using these tripeptide inhibitors, or analogues of theses as alternative or additional tools in treating the toxic effects of envenomation, as well as in thrombosis and related diseases.

## 5. Materials and Methods

### 5.1. Venoms and Venom Glands

Lyophilised crude venom was obtained from No. 1, Myanmar Pharmaceutical Factory, Yangon, Myanmar. The salivary glands from Myanmar Russell’s viper (*Daboia russelii siamensis*) were dissected in the Snake Farm, MPF, Yangon, Myanmar and used for RNA-Seq (RNA sequencing). The experimental plan was approved by the Animal Care and Use Committee, Chulalongkorn University (CU-ACUC) (No. 17/2558). Synthetic tripeptides (98% purity) were purchased from Severn Biotech Ltd., Worcestershire, UK and supplied with data from MS analysis to confirm their masses to be within 0.4% of the predicted values.

### 5.2. Transcripts Analysis of SVMPIs

Next-generation sequencing of mRNA from Myanmar Russell’s viper (2 adult males and 2 adult females) venom glands was performed on an Illumina HiSeq2000 platform. De novo assembly was performed using Trinity (r20140717). Annotation of SVMPI transcripts were archived through Blastn searches against the collected NCBI nucleotide database with search words “venom” and “serpents”. The annotations with a high score at the top hit list were picked up. 

The SVMPI transcripts were further analysed with Blastx and ORF finder for final best annotation and identification of the full-length transcript. The alignment of translated SVMPI sequences with those from other snakes were performed by using Clustal Omega followed by shading with BOXSHADE 3.21 (K. Hofmann, Koeln, Germany & M. Baron, Surrey, UK).

### 5.3. Protein Concentration

Protein concentration was determined by using Bradford reagent (BioRad, Hemel Hempstead, UK). The absorbance was measured at 595 nm and the calibration curve was prepared with a bovine gamma globulin standard (0–1.5 mg/mL). 

### 5.4. Purification of SVMPs

All chromatographic procedures were performed on either a Bio-Cad Vision Workstation (GFC) or a GE Healthcare AKTA System (anion-exchange, HIC and RP-HPLC).

#### 5.4.1. Gel Filtration Chromatography (GFC)

Crude venom (100 mg) dissolved in 5 mL of 0.01 M phosphate buffered saline (pH 7.4) was applied to a Superdex 200 column (5 × 160 cm) pre-equilibrated with the same buffer. Elution was carried out with the same buffer. The flow rate was 2 mL/min and 6 mL fractions were collected. The fractions having metalloproteinase activity (fractions 15–18) were combined for further purification. The fractions eluting near the total volume were analysed for tripeptides using RP-HPLC with subsequent MS analysis.

#### 5.4.2. Anion-Exchange Chromatography

The SVMP-containing sample obtained from GFC was applied to a Resource Q anion-exchange column (6 mL) pre-equilibrated with 0.05 M Tris-Cl buffer (pH 8.0). Elution was achieved with a linear NaCl gradient from 0 to 0.5 M in the same buffer at a flow rate of 0.6 mL/min and 1.8 mL fractions were collected. Elution was monitored at 280 nm.

#### 5.4.3. Hydrophobic Interaction Chromatography (HIC)

To further purify the SVMPs for activity measurements, fractions from Resource Q were loaded onto a Phenyl Superose column (1 mL) equilibrated in 2.5 M NaCl, 50 mM Tris-Cl, pH 7.8. Samples in Tris-Cl were adjusted to 2.5 M in NaCl and were centrifuged at 10,000× *g* for 5 min before loading onto the column. Separation was achieved by a 30 min-gradient of 2.5–0 M NaCl in 50 mM Tris-Cl, pH 7.0, using a flow rate of 0.25 mL/min. Elution was monitored at 280 nm and 0.25 mL fractions were collected.

#### 5.4.4. Reversed Phase High Performance Liquid Chromatography (RP-HPLC)

For MS analysis, SVMPs were purified using RP-HPLC rather than HIC. Fractions from Resource Q chromatography were made up to 0.2% (*v*/*v*) in TFA, centrifuged at 10,000× *g* for 5 min and then applied to Phenomenex Aeris C4 column (150 × 2.1 mm, 5 micron). The proteins were separated in a two-part acetonitrile gradient in 0.08% TFA: 0%–40% over 25 min then 40%–65% over 5 min and elution was monitored at 280 nm. The flow rate was 0.15 mL/min and 0.25 mL fractions were collected.

### 5.5. Purification of Tripeptides

Fractions from GFC suspected to contain small molecular weight components were made up to 0.2% (*v*/*v*) in TFA, centrifuged at 10,000× *g* for 5 min and applied to Phenomenex Luna C18 RP-HPLC column (100 × 2.1 mm) equilibrated in 0.08% TFA. The components were separated at 0.15 mL/min with a three-part acetonitrile gradient in 0.08% TFA: 0%–12% over 5 min, 12%–28% over 50 min and then 28%–65% over 10 min. Elution was monitored at 280 nm.

### 5.6. Mass Spectrometric Analyses

Putative RVV-X (10 μg of R1 from RP-HPLC) was reduced, treated with iodoacetamide and digested with 1.0 μg trypsin in the presence of 2 M urea. The resulting peptides were analysed by LC-ESI-MS/MS using an Acquity UPLC CSH Peptide C18 RP column (Waters, Milford, MA, USA) connected to a Q-Exactive (ThermoFisher, Northumberland, UK) MS instrument. Peaks Studio 8.0 (BSI, Waterloo, Canada) was used to analyse the resulting MS/MS data against the sequences for Eastern Russell’s viper RVV-X H chain VM3CX_DABSI (Q7LZ61) and light chains LC1 SLLC1_DABSI (Q4PRD1) and LC2 SLLC2_DABSI (Q4PRD2). 

Putative Daborhagin (5 μg of R2 from RP-HPLC) was reduced, treated with iodoacetamide and digested with 0.5 μg trypsin in the presence of 2 M urea. The resulting peptides were desalted and mass spectrometric analysis was performed using a MALDI-TOF instrument (Waters-Micromass, Milford, MA, USA). Samples were analysed by mixing a 1 μL solution of the tryptic peptides with an equal volume of 5.7 mg/mL α-cyano-4-hydroxycinnamic acid in 60% acetonitrile/0.1% trifluoroacetic acid and laying this onto a dried bed of 1 μL of 25 mg/mL α-cyano-4-hydroxycinnamic acid. Laser energy was set at 25% and detector voltage 1800 V. Ion spectra were collected in the mass range of 1000–3000 Da. Data analysis was performed using MassLynx (Waters, Milford, MA, USA). The tryptic peptide masses obtained were matched manually with those predicted (using ExPASy Peptide Mass) of a sequence for Daborhagin-K (VM3DK_DABRR; B8K1W0) retrieved from UniprotKB [27] using search word ‘Daborhagin’. 

The purified tripeptides were analysed by ESI-MS and ESI-MS/MS using the same instrument and conditions as used to analyse the tryptic peptides from RVV-X.

### 5.7. Analysis by SDS-PAGE

Protein purity was determined by SDS-PAGE [28] on a 12% or 15% resolving gel and 4% stacking gel using a Mini-PROTEAN 3 electrophoresis system (BioRad, Hemel Hemstead, UK). Samples were loaded in either reduced or non-reduced form. Gels were run at 200 V, 30 mA per gel, for 50 min. Proteins were visualised with Coomassie Brilliant Blue R250 V followed by destaining with methanol: water: acetic acid (30:60:10). Alternatively, proteins were visualised by silver staining as performed by method of Heukeshoven & Dernick [29].

### 5.8. Caseinolytic Activity

The proteolytic activity was estimated by hydrolysis of heated casein using the Anson method [30]. The reaction mixture, consisting of 500 μL casein (20 mg/mL) in 0.1 M Tris-Cl (pH 8.0), 20 μL venom was incubated for 30 min at 37 °C. The reaction was quenched by the addition of 500 μL of 5% trichloroacetic acid (TCA) at room temperature. After centrifugation at 10,000× *g* for 5 min, the hydrolysed substrate un-precipitated with TCA was determined by Folin Ciocalteau method [31]. Thus, 400 μL of the supernatant was mixed with 1 mL of 0.5M Na_2_CO_3_ and 200 μL of diluted (1:5) Folin & Ciocalteau’s phenol reagent. The mixture was then incubated at 37 °C for 30 min and the absorbance was measured at 660 nm. One enzyme unit is defined as the amount of enzyme which hydrolyses casein to produce color equivalent to 1.0 μmole of tyrosine per minute at pH 8.0 at 37 °C.

### 5.9. Gelatinolytic Activity

The gelatinolytic activity of the purified enzyme was analysed by zymography [32]. The purified metalloproteinase was diluted in SDS sample buffer under non-reducing conditions and run on 10% SDS-polyacrylamide gels (0.75 mm) co-polymerised with 0.5 mg/mL of gelatin. After electrophoresis, the gels were washed in 2.5% Triton X-100 for 30 min and then washed three times in distilled water to remove any Triton. Gels were then incubated in developing buffer (50 mM Tris-Cl, pH 7.8, 200 mM NaCl, 5 mM CaCl_2_, 0.02% Brij 35) for 18 h at 37 °C. The gels were stained with 0.5% Coomassie blue R-250 in methanol: acetic acid: water (5:10:85) solution and subsequently destained in methanol: acetic acid: water (10:5:85). The presence of gelatinolytic activity was defined as clear bands on the dark blue background.

### 5.10. Fibrinogenolytic Activity

The fibrinogenolytic activity was assayed by SDS-PAGE (4% stacking/12% resolving gel) as described by Ouyang & Teng [33]. Equal volumes of fibrinogen (1 mg/mL in 0.05 M Tris-Cl, pH 8.5) and 20 μg/mL of enzyme were incubated at 37 °C for various times intervals. At 0, 5, 15, 30, 60 and 120 min, 200 μL of the incubated solution was mixed with 400 μL of denaturing buffer containing 0.2 M Tris-Cl (pH6.8), 20% glycerol, 10% sodium dodecyl sulfate (SDS), 0.05% bromophenol blue and 10 mM β-mercaptoethanol and heated at 100 °C for 10 min to stop the digestion. Proteolytic activity was determined on the Coomassie blue-stained gel after electrophoresis by observing the cleavage patterns of purified fibrinogen chains. 

### 5.11. Inhibition of Gelatinolytic Activity

The effect of synthetic tripeptides and EDTA on purified protein was assayed using SDS-PAGE (4% stacking/10% resolving gel) to determine gelatin degradation. The purified protein (10 ng/μL) was incubated firstly with synthetic tripeptide (5 mM) or EDTA (100 μM) at 37 °C for 10 min. Then, 10 μL of gelatin solution (2 mg/mL in distilled water) was added and 20 μL of this incubated solution was taken out at 1 h and 20 h, mixed with 5 μL of 5× denaturing buffer and heated at 95 °C for 2 min. The cleavage patterns on gelatin by the enzyme was observed on Coomassie blue-stained gels after electrophoresis.

### 5.12. Inhibition of Fibrinogenolytic Activity

The effect of synthetic tripeptides or EDTA on purified protein was assayed using SDS-PAGE (4% stacking/12% resolving gel) to determine fibrinogen degradation. The purified protein (32 ng/μL) was incubated firstly with synthetic tripeptide (5 mM) or EDTA (100 μM) at 37 °C for 10 min. Then, 10 μL of fibrinogen solution (2 mg/mL in distilled water) was added and 20 μL of this incubated solution was taken out at 1 h and 20 h, mixed with 5 μL of 5× denaturing buffer and heated at 95 °C for 2 min. The cleavage effect on fibrinogen chains by the SVMPs were observed on Coomassie blue-stained gel following electrophoresis.

## Abbreviations

The following abbreviations are used in this manuscript:

SVMPs	Snake Venom Metalloproteinases
RVV-X	Russell’s Viper Venom factor X activator
NGS	Next Generation Sequencing
SVMPI	Snake Venom Metalloproteinase Inhibitor
pERW	pyroglutamate-arginine-tryptophan
pEKW	pyroglutamate-lysine-tryptophan
pENW	pyroglutamate-asparagine-tryptophan
pEQW	pyroglutamate-glutamine-tryptophan
ANP	Atrial Natriuretic Peptide
CNP	C-type Natriuretic Peptide
MRV	Myanmar Russell’s Viper
CVO	*Crotalus viridis oreganus*

## References

[1] Myint A.A., Pe T., Maw T.Z. (2002). An epidemiological study of snakebite and venomous snake survey in Myanmar. Management of Snakebite and Research; Proceedings of the Report and Working Papers of a Seminar, Yangon, Myanmar, 11–12 December 2001.

[2] Phillips R., Warrell D. (1985). Bites by Russell’s viper (*Vipera russelli siamensis*) in Burma: Haemostatic, vascular, and renal disturbances and response to treatment. Lancet.

[3] Hung D.Z., Yu Y.J., Hsu C.L., Lin T.J. (2006). Antivenom treatment and renal dysfunction in Russell’s viper snakebite in Taiwan: A case series. Trans. R. Soc. Trop. Med. Hyg..

[4] Suntravat M., Yusuksawad M., Sereemaspun A., Perez J.C., Nuchprayoon I. (2011). Effect of purified Russell’s viper venom-factor X activator (RVV-X) on renal hemodynamics, renal functions, and coagulopathy in rats. Toxicon.

[5] Mitrmoonpitak C., Chulasugandha P., Khow O., Noiprom J., Chaiyabutr N., Sitprija V. (2013). Effects of phospholipase A_2_ and metalloprotease fractions of Russell’s viper venom on cytokines and renal hemodynamics in dogs. Toxicon.

[6] Fox J.W., Serrano S.M. (2008). Insights into and speculations about snake venom metalloproteinase (SVMP) synthesis, folding and disulfide bond formation and their contribution to venom complexity. FEBS J..

[7] Markland F.S., Swenson S. (2013). Snake venom metalloproteinases. Toxicon.

[8] Gutierrez J.M., Escalante T., Rucavado A., Herrera C. (2016). Hemorrhage caused by snake venom metalloproteinases: A journey of discovery and understanding. Toxins.

[9] Kato H., Iwanaga S., Suzuki T. (1966). The isolation and amino acid sequences of new pyroglutamanylpeptides from snake venoms. Experientia.

[10] Huang K.F., Hung C.C., Wu S.H., Chiou S.H. (1998). Characterization of three endogenous peptide inhibitors for multiple metalloproteinases with fibrinogenolytic activity from the venom of Taiwan habu (*Trimeresurus mucrosquamatus*). Biochem. Biophys. Res. Commun..

[11] Huang K.F., Chiou S.H., Ko T.P., Wang A.H.J. (2002). Determinants of the inhibition of a Taiwan habu venom metalloproteinase by its endogenous inhibitors revealed by x-ray crystallography and synthetic inhibitor analogues. Eur. J. Biochem..

[12] Francis B., Kaiser I.I. (1993). Inhibition of metalloproteinases in *Bothrops asper* venom by endogenous peptides. Toxicon.

[13] Munekiyo S.M., Mackessy S.P. (2005). Presence of peptide inhibitors in rattlesnake venoms and their effects on endogenous metalloproteases. Toxicon.

[14] Wagstaff S.C., Favreau P., Cheneval O., Laing G.D., Wilkinson M.C., Miller R.L., Stocklin R., Harrison R.A. (2008). Molecular characterisation of endogenous snake venom metalloproteinase inhibitors. Biochem. Biophys. Res. Commun..

[15] Ding B., Xu Z., Qian C., Jiang F., Ding X., Ruan Y., Ding Z., Fan Y. (2015). Antiplatelet aggregation and antithrombosis efficiency of peptides in the snake venom of *Deinagkistrodon acutus*: Isolation, identification, and evaluation. Evid. Based Complement. Alternat. Med..

[16] Chen H.-S., Tsai H.-Y., Wang Y.-M., Tsai I.-H. (2008). P-III hemorrhagic metalloproteinases from Russell’s viper venom: Cloning, characterization, phylogenetic and functional site analyses. Biochimie.

[17] Calvete J.J., Juarez P., Sanz L. (2007). Snake venomics. Strategy and applications. J. Mass Spectrom..

[18] Tan N.H., Fung S.Y., Tan K.Y., Yap M.K., Gnanathasan C.A., Tan C.H. (2015). Functional venomics of the Sri Lankan Russell’s viper (*Daboia russelii*) and its toxinological correlations. J. Proteom..

[19] Gowda D.C., Jackson C.M., Hensley P., Davidson E.A. (1994). Factor X-activating glycoprotein of Russell’s viper venom. J. Biol. Chem..

[20] Takeya H., Nishida S., Miyata T., Kawada S.-I., Saisaka Y., Morita T., Iwanaga S. (1992). Coagulation factor X activating enzyme from Russell’s viper venom (RVV-X). J. Biol. Chem..

[21] Chen H.S., Chen J.M., Lin C.W., Khoo K.H., Tsai I.H. (2008). New insights into the functions and *N*-glycan structures of factor X activator from Russell’s viper venom. FEBS J..

[22] Siigur E., Tonismagi K., Trummal K., Samel M., Vija H., Subbi J., Siigur J. (2001). Factor X activitor from *Vipera lebetina* snake venom, molecular characterization and substrate specificity. Biochim. Biophys. Acta.

[23] Yee K.T., Khow O., Noiphrom J., Kyaw A.M., Maw L.Z., Kyaw M.T., Chulasugandha P. (2014). Purification and characterization of metalloproteinase from Myanmar Russell’s viper (*Vipera russelii*) venom. Myanmar Health Sci. Res. J..

[24] Risch M., Georgieva D., von Bergen M., Jehmlich N., Genov N., Arni R.K., Betzel C. (2009). Snake venomics of the Siamese Russell’s viper (*Daboia russelli siamensis*)—Relation to pharmacological activities. J. Proteom..

[25] Fischer W.H., Spiess J. (1987). Identification of a mammalian glutaminyl cyclase converting glutaminyl into pyroglutamyl peptides. Proc. Natl. Acad. Sci. USA.

[26] Varro A. (2007). Posttranslational processing: Peptide hormones and neuropeptide transmitters. eLS.

[27] UniprotKB. http://www.uniprot.org.

[28] Laemmli U.K. (1970). Cleavage of structural proteins during the assembly of the head of bacteriophage T4. Nature.

[29] Heukeshoven J., Dernick R. (1985). Simplified method for silver staining of proteins in polyacrylamide gels and the mechanism of silver staining. Electrophoresis.

[30] Anson M.L. (1938). The estimation of pepsin, trypsin, papain, and cathepsin with hemoglobin. J. Gen. Physiol..

[31] Folin O., Ciocalteu V. (1927). On tyrosine and tryptophane determinations in proteins. J. Biol. Chem..

[32] Toth M., Fridman R., Brooks S.A., Schumacher U. (2001). Assessment of gelatinases (MMP-2 and MMP-9) by gelatin zymography. Metastasis Research Protocols.

[33] Ouyang C., Teng C.-M. (1976). Fibrinogenolytic enzymes of *Trimeresurus macrosquamatus* venom. Biochim. Biophys. Acta.

